# Isolation and identification of *Xenorhabdus* and *Photorhabdus* bacteria associated with entomopathogenic nematodes and their larvicidal activity against *Aedes aegypti*

**DOI:** 10.1186/s13071-017-2383-2

**Published:** 2017-09-21

**Authors:** Chamaiporn Fukruksa, Thatcha Yimthin, Manawat Suwannaroj, Paramaporn Muangpat, Sarunporn Tandhavanant, Aunchalee Thanwisai, Apichat Vitta

**Affiliations:** 1 Department of Microbiology and Parasitology, Faculty of Medical Science, Naresuan University, Phitsanulok, 65000 Thailand; 2 Department of Microbiology and Immunology, Faculty of Tropical Medicine, Mahidol University, Bangkok, 10400 Thailand; 3 Centre of Excellence in Medical Biotechnology (CEMB), Faculty of Medical Science, Naresuan University, Phitsanulok, 65000 Thailand; 4 Center of Excellence for Biodiversity, Faculty of Sciences, Naresuan University, Phitsanulok, 65000 Thailand

**Keywords:** *Xenorhabdus*, *Photorhabdus*, Phylogeny, Larvicidal activity, *Aedes aegypti*

## Abstract

**Background:**

*Aedes aegypti* is a potential vector of West Nile, Japanese encephalitis, chikungunya, dengue and Zika viruses. Alternative control measurements of the vector are needed to overcome the problems of environmental contamination and chemical resistance. *Xenorhabdus* and *Photorhabdus* are symbionts in the intestine of entomopathogenic nematodes (EPNs) *Steinernema* spp. and *Heterorhabditis* spp. These bacteria are able to produce a broad range of bioactive compounds including antimicrobial, antiparasitic, cytotoxic and insecticidal compounds. The objectives of this study were to identify *Xenorhabdus* and *Photorhabdus* isolated from EPNs in upper northern Thailand and to study their larvicidal activity against *Ae. aegypti* larvae.

**Results:**

A total of 60 isolates of symbiotic bacteria isolated from EPNs consisted of *Xenorhabdus* (32 isolates) and *Photorhabdus* (28 isolates). Based on *recA* gene sequencing, BLASTN and phylogenetic analysis, 27 isolates of *Xenorhabdus* were identical and closely related to *X. stockiae*, 4 isolates were identical to *X. miraniensis*, and one isolate was identical to *X. ehlersii*. Twenty-seven isolates of *Photorhabdus* were closely related to *P. luminescens akhurstii* and *P. luminescens hainanensis,* and only one isolate was identical and closely related to *P. luminescens laumondii*. *Xenorhabdus* and *Photorhabdus* were lethal to *Ae aegypti* larvae. *Xenorhabdus ehlersii* bMH9.2_TH showed 100% efficiency for killing larvae of both fed and unfed conditions, the highest for control of *Ae. aegypti* larvae and *X. stockiae* (bLPA18.4_TH) was likely to be effective in killing *Ae. aegypti* larvae given the mortality rates above 60% at 72 h and 96 h.

**Conclusions:**

The common species in the study area are *X. stockiae*, *P. luminescens akhurstii,* and *P. luminescens hainanensis*. Three symbiotic associations identified included *P. luminescens akhurstii-H. gerrardi*, *P. luminescens hainanensis-H. gerrardi* and *X. ehlersii-S. Scarabaei* which are new observations of importance to our knowledge of the biodiversity of, and relationships between, EPNs and their symbiotic bacteria. Based on the biological assay, *X. ehlersii* bMH9.2_TH begins to kill *Ae. aegypti* larvae within 48 h and has the most potential as a pathogen to the larvae. These data indicate that *X. ehlersii* may be an alternative biological control agent for *Ae. aegypti* and other mosquitoes.

## Background

Dengue fever (DF) caused by dengue virus is the most important vector-borne disease transmitted by *Aedes* spp. In southeast Asia, including Thailand, the primary vector for dengue virus is *Aedes aegypti* and the secondary vector is *Ae. albopictus* [1]. Dengue fever is a major risk to public health due to the recent spread of the virus worldwide [2, 3]. This disease threatens approximately 390 million people living in over 100 countries [4, 5]. *Aedes* spp. are not only the vectors of the dengue virus but also of West Nile virus and chikungunya virus [6]. West Nile virus infects more than 2.5 million people causing over 1300 deaths during 1999–2010 [7]. Chikungunya virus normally does not cause death in humans but has been reported in more than 45 countries with epidemic in India [8]. Recently, the Zika virus, a worldwide public health concern, was shown to be transmitted to humans by *Ae. aegypti* [4]. More than 200,000 human cases infected with the Zika virus have been reported in the Americas. Approximately 3.6 billion people worldwide are living in at-risk areas for Zika virus, dengue virus, chikungunya, and yellow fever virus transmission [9]. The epidemics of Zika virus and dengue virus are the greatest public health threats to the human population worldwide. There are no specific treatments or vaccines currently available to combat infections by these viruses; the only effective approach to prevent infection is to avoid mosquito bites [10]. Therefore, control of the larval and adult mosquitoes is an essential precautionary measure to prevent the disease. To reduce mosquito population density, life span and human contact, control measures against *Aedes* include elimination of breeding sites, use of chemical control, genetic and biological control [11]. Elimination of breeding places is a simple technique and low-cost method to protect the breeding sites of *Aedes*. Chemical control (organochlorides, DDT; organophosphates, OP; pyrethroids) is usually considered as the first method for mosquito control. However, repeated use of pesticides leads to development of insecticide-resistant mosquito populations and toxic to humans. *Aedes* spp. in all regions of the world have developed resistance to DDT [12]. Pyrethroid resistance in mosquitoes was also reported in several countries in Asia [12]. In addition, the insecticide also causes environmental contamination and destruction of non-target organisms. Genetic control including the sterile insect technique (SIT) and rearing of insects carrying a dominant lethal allele (RIDL) are species-specific methods for *Ae. albopictus* and *Ae. aegypti* [13, 14]. However, most of the genetic control methods are in the laboratory conditions, and need more consideration in several aspects such as cost, natural condition and environmental risk assessment [11]. Larval control is the most economical method to eradicate *Aedes* spp. This method can scope on a certain source with or without pesticide applications rather than spraying miles of chemicals for control of the adult stage.

Biological control is an alternative method used for mosquito vectors. Compared to chemical control it is environmentally friendly as well as sustainable because of the slow pace of resistance development against biological control agents. In addition, the continued use of chemicals over a long period of time can induce insecticide resistance in mosquito populations. The biological control agents used against *Aedes* mosquitoes include *Bacillus thuringiensis israelensis* or *B. sphericus* and their toxins and *Xenorhabdus*/*Photorhabdus* [15–19], fungi *Metarhizium anisopliae* and *Beauveria bassiana* [20–22], the protozoan *Acanthamoeba polyphaga* [23] and the copepod *Macrocyclops albidus* [24]. It has been suggested that *Bacillus thuringiensis*, *Xenorhabdus* and *Photorhabdus* have potential for the biological control of *Aedes* mosquitoes. These entomopathogenic bacteria are used in the control of mosquito larvae [17, 25].

*Xenorhabdus* and *Photorhabdus*, Gram-negative bacteria of the family Enterobacteriaceae, are symbiotic bacteria with entomopathogenic nematodes (EPNs) of the genera *Steinernema* and *Heterorhabditis*, respectively [26]. These bacteria produce many bioactive compounds which demonstrate insecticidal, cytotoxic and anti-microbial activities [27]. *Xenorhabdus* and *Photorhabdus* live in the gut of the infective juvenile stages of EPNs. They can enter the insect hosts by the aid of EPNs through a natural orifice such as the mouth and anus or by direct penetration through the skin. The bacteria are released into the blood system of the insects and then multiply, release toxins and antimicrobial compounds which result in insect septicemia and death within 24–48 h [27]. Symbiotically the EPNs benefit from living with the bacteria by eating the bacteria and the remains of the infected insects. The EPNs grow and reproduce, increasing the number of infective insect juvenile EPNs which then leave the host insect cadaver to find a new host.

*Xenorhabdus* and *Photorhabdus* have been successfully used to reduce the development of several insect pests in laboratory conditions [28]. The use of *P. luminescens* mixed with *Bacillus thuringiensis kurstaki* inhibits the growth of the African cotton leafworm *Spodoptera littoralis* (Lepidoptera: Noctuidae) [29]. Forst et al. [30] reported that 20 cells of *X. nematophilus* killed as much as 90% of the larvae of *Manduca sexta*. The *Pir*AB toxins from *P. asymbiotica* exhibited potential for reduction the survival of the larvae of *Ae. aegypti* and *Ae. albopictus* larvae. An oral dose of *X. nematophila* and *P. luminescens* cell suspension is lethal to *Aedes* larvae even in the absence of the entomopathogenic nematode [25].

The first survey of symbiotic bacteria in Thailand demonstrated that *Xenerhabdus* and *Photorhabdus* are found throughout the country with predomination of *X. stockiae* and *P. luminescens* [31]. Subsequently it was found that *Xenorhabus* sp. PB61.4 isolated from EPN from Chaiyaphum Province in the northeast Thailand are able to produce a novel substance (chaiyaphumine) that is toxic to *Plasmodium falciparum* in vitro [32]. Thus, Thailand is a natural environment for studying *Xenorhabdus* and *Photorhabdus* diversity and for identifying their potential bioactive compounds. We previously isolated and identified the EPNs collected from upper northern Thailand [33]. The present study continues our work on the identification of symbiotic bacteria. Our objectives were to identify *Xenorhabdus* and *Photorhabdus* isolated from entomopathogenic nematodes in the upper northern regions of Thailand and to study their larvicidal activity against *Ae. aegypti* larvae in laboratory conditions. Here we report the identification and phylogenetic analysis of symbiotic bacteria based on sequencing of the housekeeping gene (*recA*) which gave more reliable results for the taxonomy purposes. In addition, phylogenetic analyses of the *recA* gene showed the potential topology for distinguishing between *Xenorhabdus* spp. and *Photorhabdus* spp.

## Methods

### Isolation and identification of *Xenorhabdus* and *Photorhabdus*

Eight provinces located in the upper northern Thailand including Chiang Mai, Chiang Rai, Nan, Phayao, Phrae, Lampang, Lamphun, and Mae Hong Son were selected for soil sampling. *Xenorhabdus* and *Photorhabdus* bacteria were isolated from the hemolymph of larval *Galleria mellonella* (greater wax moth) cadavers infected with EPNs that were previously isolated and identified as described in Vitta et al. [33]. To propagate EPNs and obtain hemolymphs containing the symbiotic bacteria, water containing approximately 300 EPNs (500 μl) was placed onto a sterile Petri dish containing 5 larvae of *G. mellonella*. The Petri dish was then sealed with parafilm and incubated in the dark at room temperature. The insect larvae were observed daily for 2–3 days. The resulting insect cadavers were then washed with 95–100% ethanol and placed on another sterile Petri dish. The third segment from the mouth parts of the dead *G. mellonella* larvae were opened using fine sterile forceps to obtain the hemolymph containing *Xenorhabdus* and *Photorhabdus*. A drop of hemolymph was streaked on sterile plates of nutrient bromothymol blue-triphenyltetrazolium chloride agar (NBTA) which were then stored in the dark at room temperature. After 4 days of incubation, preliminary identification of these bacteria was performed by observing the colony morphology. The colonies of species of the genus *Xenorhabdus* are dark blue, convex, umbonated and swarm while those of species of the genus *Photorhabdus* are dark green, convex and umbonated [31]. A single colony from each isolate was subcultured on the same medium and kept in Luria-Bertani (LB) broth supplemented with 20% glycerol at -80 °C for further species identification and bioassay.

Species identification of *Xenorhabdus* and *Photorhabdus* was carried out by polymerase chain reaction (PCR) and analysis of partial *rec*A gene sequences. Genomic DNA from each bacterial isolate extracted using a genomic DNA mini kit (Geneaid Biotech Ltd., New Taipei, Taiwan). PCR primers [34], reagents and PCR amplication conditions used were as described in Thanwisai et al. [31]. The PCR cycles were conducted in an Applied Biosystems thermal cycler (Life Technologies, Carlsbad, CA, USA). The PCR products were purified using a Gel/PCR DNA Fragment Extraction Kit (Geneaid Biotech Ltd., New Taipei, Taiwan) before sequencing at Macrogen Inc., Korea. All sequences were edited using the SeqManII program (DNASTAR inc., Wisconsin, Madison, USA). A BLASTN search was performed to identify *Xenorhabdus* and *Photorhabdus* to the species level by finding the similarity of the *rec*A sequences with known sequences in the NCBI database (http://blast.ncbi.nlm.nih.gov/Blast.cgi).

### Phylogenetic analysis of *Xenorhabdus* and *Photorhabdus*

Phylogenetic analysis was performed using MEGA version 5.2 [35]. Twenty three *recA* sequences for *Xenorhabdus* spp. and 15 sequences for *Photorhabdus* spp. were downloaded from the NCBI database; *Escherichia coli* (GenBank: U00096.3) was used as out-group. Multiple sequences were aligned using Clustal W version 5.2 for editing the nucleotide sequences which were trimmed to 508 bp. Maximum likelihood trees were costructed using the Kimura-2 model (1000 bootstrap replicates).

### Biological assay

A total of 12 bacterial isolates of *Xenorhabdus* (8 isolates) and *Photorhabdus* (4 isolates) were randomly selected from 60 isolates based on different branches in a maximum-likelihood tree.

The eight *Xenorhabdus* isolates were divided into 3 groups: (i) *Xenorhabdus* bMH9.2_TH isolate closely related to *X. ehlersii*; (ii) *Xenorhabdus* bMH16.4_TH, bMH16.1_TH, bMH4.5_TH isolates closely related to *X. miraniensis*; (iii) *Xenorhabdus* bLPA12.2_TH, bLPA18.4_TH, bCR7.3_TH and bPH23.5_TH isolates closely related to *X. stockiae*. The *Photorhabdus* isolates were divided into 3 groups: (i) *Photorhabdus* bPY17.4_TH and bLPO16.2_TH closely related to *P. luminescens hainanensis*; (ii) *Photorhabdus* bNA22.1_TH closely related to *P. luminescens akhurstii*; and (iii) *Photorhabdus* bMH8.4_TH closely related to *P. luminescens laumondii*. All selected *Xenorhabdus* and *Photorhabdus* isolates (tested group) were separately grown on NBTA. *Escherichia coli* ATCC®25922 cultured on Tryptone soy agar (TSA), was used as one negative control and distilled water was used as a second negative control.

A single colony of each isolate of *Photorhabdus* and *Xenorhabdus* on NBTA was transferred, in sterile conditions, into a 15 ml tube containing 5 ml of 5YS broth medium which was composed of 5% yeast extract (w/v), 0.5% NaCl (w/v), 0.05% K_2_HPO_4_ (w/v), 0.05% NH_2_H_2_PO_4_ (w/v), 0.02% MgSO_4_.7H_2_O (w/v) [36]. The tubes were then incubated while shaking (150 *rpm*) at room temperature for 48 h. Each tube of the bacterial suspension was then centrifuged at 10,000 *rpm* for 10 min to obtain a bacterial pellet which was then resuspended in sterile distilled water. Adjustment of the bacterial suspension at OD_600_ to 1.0 was performed by spectrophotometer (BECMAN-COUTER Model DU®730, Fullerton, USA). A single colony of *E. coli* ATCC®25922 on TSA was subcultured on 5YS broth and then processed under the same conditions applied for *Photorhabdus* and *Xenorhabdus*. A 10-fold serial dilution spread plate was performed and the concentration of bacterial suspension was found to be 10^8^ (CFU/ml).

*Aedes aegypti* third- and fourth-instar larvae were purchased from the Medical Entomology Division of the National Institute of Health, Department of Medical Sciences, Ministry of Public Health of Thailand. The larvae were transported to the Department of Microbiology and Parasitology at the Faculty of Medical Science, Naresuan University. *Aedes aegypti* larvae were rested in dechlorinated water for one day prior to test.

A biological assay was performed under two conditions; fed condition (larvae exposed to symbiotic bacteria were fed with ground pet food) and unfed condition (larvae exposed to symbiotic bacteria were not given food). *Escherichia coli* ATCC®25922 and distilled water were used as negative controls. For each bioassay, 30 larvae (10 larvae/well) were transferred to 3 wells of a 24-well microtiter plate. Two ml bacterial suspension of each isolate containing 10^8^ CFU/ml was added in to each well. The plates were kept at room temperature, and the larval mortality was observed daily for 4 days (24, 48, 72 and 96 h). Larvae that showed no movement response after teasing with a fine sterile toothpick were considered dead. All assays were carried out 3 times.

### Statistical analysis

The mortality rate (percentages) of the larvae in the control groups and in the treatment group (each bacterial isolate) were statistically tested using SPSS version 17 (Fisher’s exact test; *P* < 0.05). Fisher’s exact test was performed to determine the difference in mortality rates between the fed and unfed groups.

## Results

### Identification of *Xenorhabdus* and *Photorhabdus*

Based on the colony morphology on the NBTA, 32 isolates of *Xenorhabdus* were isolated from the EPNs and were preliminarily characterized based on a dark blue, covex and umbonated or swarm colony, while colonies of *Photorhabdus* (28 isolates) were dark green, convex and umbonated.

A partial region of the *rec*A gene from both *Xenorhabdus* and *Photorhabdus* was determined by PCR and sequenced. PCR amplicons of the *rec*A gene of *Xenorhabdus* and *Photorhabdus* were approximately 900 bp in size. After BLASTN search, 27 isolates of *Xenorhabdus* (GenBank: KY404017–KY404048) showed sequence similarity to *X. stockiae* (97–99% similarity), while 4 isolates to *X. miraniensis* (98–100% similarity) and only one isolate of *Xenorhabdus* was recognized as *X. ehlersii* with 97% similarity. For *Photorhabdus* (GenBank: KY436924–KY436951), 18 isolates, 9 isolates and 1 isolate showed sequence similarity to *P. luminescens akhurstii* (98–100% similarity), *P. luminescens hainanensis* (98–99% similarity), and *P. luminescens laumondii* (98% similarity), respectively.

### Phylogenetic tree of *Xenorhabdus* and *Photorhabdus*

**Fig. 1:**
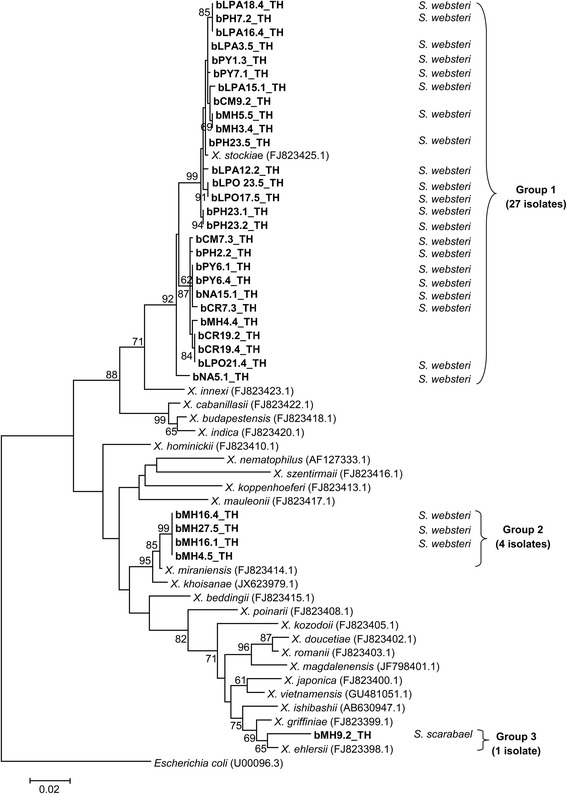
Phylogenetic relationships of *Xenorhabdus* (*n* = 32) isolated from EPNs of upper northern Thailand and *Xenorhabdus* spp. worldwide based on maximum-likelihood analysis of 508 bp of the *recA* region. Bootstrap values are based on 1000 pseudoreplicates. Codes for bacterial isolates indicated in the phylogenetic tree are defined as province/soil-site collection/country, e.g. bMH9.2_TH (b, bacteria; MH, Mae Hong Son Province; 9.2, soil collection site; TH, Thailand). Eight provinces located in the upper northern Thailand including Chiang Mai (CM), Chiang Rai (CR), Nan (NA), Phayao (PY), Phrae (PH), Lampang (LPA), Lamphun (LPO), and Mae Hong Son (MH) were selected for soil sampling

**Fig. 2:**
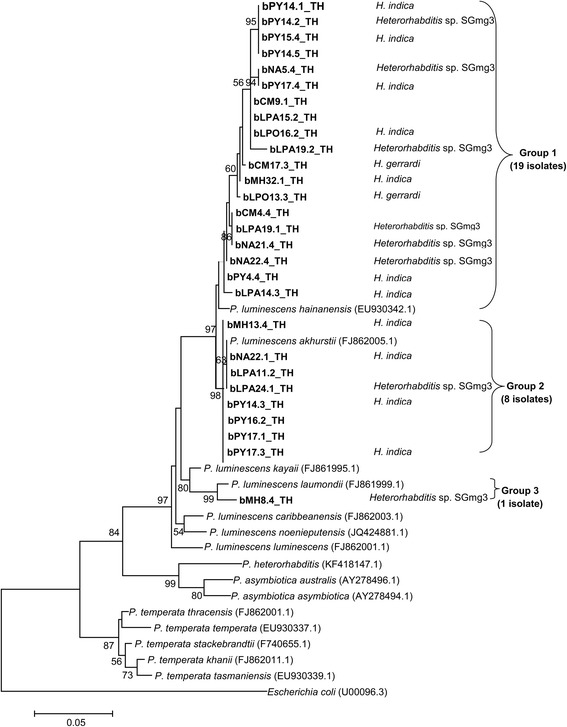
Phylogenetic relationships of *Photorhabdus* (*n* = 28) isolated from EPNs of upper northern Thailand and *Photorhabdus* spp. worldwide based on maximum-likelihood analysis of 508 bp of the *recA* region. Bootstrap values are based on 1000 pseudoreplicates. Codes for bacterial isolates indicated in the phylogenetic tree are defined as province/soil-site collection/country, e.g. bMH9.2_TH (b, bacteria; MH, Mae Hong Son Province; 9.2, soil collection site; TH, Thailand). Eight provinces located in the upper northern Thailand including Chiang Mai (CM), Chiang Rai (CR), Nan (NA), Phayao (PY), Phrae (PH), Lampang (LPA), Lamphun (LPO), and Mae Hong Son (MH) were selected for soil sampling

Based on the maximum-likelihood tree based on 508 bp of the *rec*A region, most *Xenorhabdus* isolates (27 isolates) in Group 1 were closely related to the *X. stockiae* strain TH01 (GenBank: FJ823425.1). All identified isolates of *X. stockiae* were associated with *Steinernema websteri*. Four isolates of *Xenorhabdus* (Group 2) grouped with *X. miraniensis* (GenBank: FJ823414.1). All identified isolates of *X. miraniensis* were hosted by *Steinernema websteri*. The remaining *Xenorhabdus* isolate (Group 3) was clustered in *X. ehlersii* (GenBank: FJ823398.1). Unlike the other two clusters, this *Xenorhabdus* isolate was associated with *Steinernema scarabaei* (Fig. 1)*.* From the phylogenetic analysis of *Photorhabdus*, two main groups (Group 1 and 2) containing most of the isolates of *Photorhabdus* (27 isolates) were clustered into a group containing *P. luminescens akhurstii* (GenBank: EU862005.1) and *P. luminescens hainanensis* (GenBank: EU930342.1) (Fig. 2). Most *P. luminescens akhurstii* isolates (8 isolates) were associated with *Heterorhabditis indica*. One isolate each of *P. luminescens akhurstii* (bCM17.3_TH) and *P. luminescens hainanensis* (bLPO13.3_TH) were hosted by *Heterorhabditis gerrardi*. The group with only one isolate of *Photorhabdus* (Group 3) was grouped together with *P. luminescens laumondii* (GenBank: FJ861999.1) which was associated with *Heterorhabditis* sp. SGmg3 (Fig. 2).

### Larvicidal activity of *Xenorhabdus* and *Photorhabdus* against *Aedes aegypti*

*Xenorhabdus ehlersii* (bMH9.2_TH) showed highest effectiveness for killing *Aedes aegypti* larvae under both fed and unfed conditions. The mortality rates of *Ae. aegypti* larvae in fed condition were 39%, 96%, 98%, and 100% after exposure to a suspension of *X. ehlersii* (bMH9.2 TH) for 24, 48, 72 and 96 h, respectively, while the mortality rates of unfed larvae were 56%, 98%, 99% and 100% at similar time intervals (Table 1). These rates were significantly different (Fisher’s exact test, *P* < 0.05) when compared with the mortality rates of *Ae. aegypti* larvae in the control groups which were 1% and 2% for *Escherichia coli* ATCC®25922 and distilled water in fed condition, and 7% and 0% for *E. coli* ATCC® 25,922 and distilled water in unfed condition (Table 1). *Xenorhabdus stockiae* (bLPA18.4_TH) is likely to be effective in killing *Ae. aegypti* larvae given the mortality rates above 60% at 72 and 96 h under both fed and unfed condition. However, the other isolates of *X. stockiae* (bLPA12.2_TH, bCR7.3_TH and bPH23.5_TH) were less pathogenic to the larvae, exhibiting a mortality rates lower than 50%. All isolates of *X. miraniensis* (bMH16.4_TH, bMH16.1_TH and bMH4.5_TH) showed a negligible toxicity to *Ae. aegypti* larvae and the observed mortality rate was lower than 20%. All *Photorhabdus* isolates (bPY17.4_TH, bLPO16.2_TH, bMH8.4_TH and bNA22.1_TH) showed low pathogenic effect on *Ae. aegypti* larvae upon oral uptake, with a mortality rate lower than 40% (Table 1). The mortality rates were significantly different between fed and unfed mosquito larvae in case of *P. luminescens hainanensis* (bLPO16.2_TH) and *X. stockiae* (bCR7.3_TH and bPH23.5_TH) (see Table 2).

**Table 1:** Mortality rates of *Aedes aegypti* larvae after exposure to *Xenorhabdus* and *Photorhabdus* in fed and unfed condition

Bacterium (Code)	Mortality rate (%)							
	Fed condition				Unfed condition			
	24 h	48 h	72 h	96 h	24 h	48 h	72 h	96 h
*Xenorhabdus ehlersii* (bMH9.2_TH)	39*	96*	98*	100*	56*	98*	99*	100*
*Xenorhabdus miraniensis* (bMH16.4_TH)	7	9	18*	18	0^nd^	0	6	6
*Xenorhabdus miraniensis* (bMH16.1_TH)	0^nd^	3	3	6	0^nd^	6	8*	8*
*Xenorhabdus miraniensis* (bMH4.5_TH)	3	7*	9*	12*	3	3	7	20*
*Xenorhabdus stockiae* (bLPA12.2_TH)	4	14*	41*	43*	3	20*	27*	37*
*Xenorhabdus stockiae* (bLPA18.4_TH)	16*	54*	64*	67*	24*	47*	67*	67*
*Xenorhabdus stockiae* (bCR7.3_TH)	0^nd^	1	2	6*	0^nd^	0	1	23*
*Xenorhabdus stockiae* (bPH23.5_TH)	0^nd^	4	7	7	3	7	9*	33*
*Photorhabdus luminescens hainanensis* (bPY17.4_TH)	2	7	21*	21*	2	4	9*	9*
*Photorhabdus luminescens hainanensis* (bLPO16.2_TH)	1	12*	14*	21*	1	1	4	19*
*Photorhabdus luminescens laumondii* (bMH8.4_TH)	1	20*	26*	33*	2	7	9*	19*
*Photorhabdus luminescens akhurstii* (bNA22.1_TH)	4	14*	22*	24*	4	14*	19*	21*
Control: *Escherichia coli* ATCC®25900	0	0	1	1	0	5	7	7
Control: Distilled water	0	0	2	2	0	0	0	0

*Significant difference (*P* < 0.05) among symbiotic bacteria and controls by Fisher’s exact test

*Abbreviation*: *nd* not determined

**Table 2:** Comparative data for mortality rates of fed and unfed *Aedes aegypti*

Bacterium (Code)	Mortality rate (%)		*P*	Mortality rate (%)		*P*	Mortality rate (%)		*P*	Mortality rate (%)		*P*
	24 h			48 h			72 h			96 h		
	Condition			Condition			Condition			Condition		
	Fed	Unfed		Fed	Unfed		Fed	Unfed		Fed	Unfed	
*Xenorhabdus ehlersii* (bMH9.2_TH)	39	56	0.260	96	98	1.000	98	99	1.000	100	100	nd
*Xenorhabdus miraniensis* (bMH16.4_TH)	7	0	0.471	9	0	0.206	18	6	0.251	18	6	0.251
*Xenorhabdus miraniensis* (bMH16.1_TH)	0	0	nd	3	6	0.135	3	8	0.082	6	8	0.110
*Xenorhabdus miraniensis* (bMH4.5_TH)	3	3	0.206	7	3	1.000	9	7	1.000	12	20	0.051
*Xenorhabdus stockiae* (bLPA12.2_TH)	4	3	1.000	14	20	0.712	41	27	0.087	43	37	0.899
*Xenorhabdus stockiae* (bLPA18.4_TH)	16	24	0.931	54	47	0.357	64	67	0.534	67	67	1.000
*Xenorhabdus stockiae* (bCR7.3_TH)	0	0	nd	1	0	1.000	2	1	1.000	6	23	0.030*
*Xenorhabdus stockiae* (bPH23.5_TH)	0	3	0.471	4	7	1.000	7	9	0.436	7	33	0.022*
*Photorhabdus luminescens hainanensis* (bPY17.4_TH)	2	2	0.765	7	4	0.316	21	9	0.074	21	9	0.074
*Photorhabdus luminescens hainanensis* (bLPO16.2_TH)	1	1	1.000	12	1	0.029*	14	4	0.103	21	19	1.000
*Photorhabdus luminescens laumondii* (bMH8.4_TH)	1	2	1.000	20	7	0.499	26	9	0.572	33	19	0.893
*Photorhabdus luminescens akhurstii* (bNA22.1_TH)	4	4	0.620	14	14	0.364	22	19	1.000	24	21	1.000

*Significant difference (*P* < 0.05) between fed and unfed condition by Fisher’s exact test

*Abbreviation*: *nd* not determined

## Discussion

*Xenorhabdus stockiae* and *P. luminescens akhurstii* were the most common symbiotic bacteria that we found in EPNs of upper northern Thailand. Most *X. stockiae* isolates were associated with *S. websteri* while *P. luminescens akhurstii* was associated with *H. indica* [33]. This is consistent with a previous report showing that 66 isolates of *X. stockiae* and three isolates of *X. miraniensis* were isolated from *S. websteri* and 57 isolates of *P. luminescens hainanensis* and a few isolates of *P. luminescens laumondii* and *P. luminescens akhurstii* were associated with *H. indica* [31]. This suggests that the common association in the study area is *X. stockiae* - *S. websteri* and *P. luminescens akhurstii* - *H. indica*.

Two *Photorhabdus* isolates, *P. luminescens akhurstii* (bCM17.3_TH) and *P. luminescens hainanensis* (bLPO13.3_TH), were associated with *Heterorhabditis gerrardi*. *Photorhabdus luminescens akhurstii* was previously reported to be in a symbiotic association with *H. indica* [34]. This suggests that *P. luminescens akhurstii* can be hosted by not only *H. indica* but also by *H. gerrardi*. The EPN *H. gerrardi* was previously reported as being associated with *P. asymbiotica* [37]. *Photorhabdus luminescens akhurstii* and *H. gerrardi* is a new EPN-symbiotic bacteria association. *Photorhabdus luminescens hainanensis* were hosted by *Heterorhabditis* sp. in China [34]*.* To our knowledge, this is the first record of the association between *P. luminescens hainanensis* and *H. gerrardi*. In our study, one isolate of *X. ehlersii* was shown to be associated with *S. scarabaei*. Previous studies reported that *X. ehlersii* is associated with *S. serratum* [38] and *S. longicaudum* [39] while *S. sarabaei* is associated with *X. koppenhoeferi* [40]. The symbiotic relationship of *X. ehlersii* - *S. scarabaei* complex is a novel association observed and reported in our study.

Phylogenetic analysis of 32 isolates of *Xenorhabdus* showed that most *Xenorhabdus* isolates are closely related to *X. stockiae* which is similar to previous reports [31]. A small number of *Xenorhabdus* isolates were closely related to *X. miraniensis* and only one isolate was closely related to *X. ehlersii* isolated from China [38]. In our study we found *X. stockiae* as the majority of the isolates of the genus *Xenorhabdus*. Most of the *Photorhabdus* isolates were closely related to *P. luminescens akhurstii* and *P. luminescens hainanensis*. *P. luminescens akhurstii* was predominant among the isolates of the genus *Photorhabdus*. Three new associations between EPN and symbiotic bacteria which were observed in this study: *H. gerrardi* - *P. luminescens akhurstii*, *H. gerrardi* - *P. luminescens hainanensis* and *S. scarabaei* - *X. ehlersii* complexes.

*Xenorhabdus ehlersii* bMH9.2_TH killed up to 100% of *Ae. aegypti* at 96 h, in both fed and unfed conditions. Previous studies on the same group of symbiotic bacteria reported that a cell suspension of *P. luminescens* kills up to 73% of *Ae. aegypti* larvae in fed groups, and 83% in unfed groups, followed by *X. nematophila* with larval mortality of up to 52% in the fed condition and 42% in the unfed condition [25]. This may be due to *X. ehlersii* bMH9.2_TH producing bioactive compounds that are effective in killing mosquito larvae. XeGroEL protein produced from *X. ehlersii* has been proven to kill *Galleria mellonella* [41, 42]. In addition, other species of *Xenorhabdus* such as *X. nematophila* can produce compounds with insecticidal properties such as toxin complexes or lipopolysaccharides [43–45]. All compounds mentioned above were suggested to play roles as insecticidal compounds. In addition, *X. stockiae* (bLPA18.4_TH) in this study was considered an effective entomopathogen to kill *Ae. aegypti* larvae. *Xenorhabdus stockiae* PB09 was previously reported to have acaricidal and antibacterial activities [46, 47].

Thus our study is the first report of insecticidal activity of *X. stockiae*. This suggests that *X. stockiae* may produce several bioactive compounds as it manifests several bioactivities: however, there are no reports on purified bioactive compounds from *X. stockiae*. Bioactive compounds from *X. stockiae* isolates should be further investigated for better understanding of their mechanism of action. Unlike *X. stockiae* (bLPA18.4_TH), other *X. stockiae* isolates (bLPA12.2_TH, bCR7.3_TH and bPH23.5_TH) showed lower pathogenicity against *Ae. aegypti* larvae as indicated by the low mortality rates. This may be due to differences in the ability to produce bioactive compounds among different *X. stockiae* isolates. In this study, we cannot conclude that any substance resulted in the death of *Ae. aegypti* larvae, but *Xenorhabdus* and *Photorhabdus* showed larvicidal activity by oral toxicity against *Ae. aegypti*. In addition, the mechanisms of *Xenorhabdus* and *Photorhabdus* for killing *Ae. aegypti* larvae are difficult to explain. The isolation of compounds produced by *X. ehlersii* bMH9.2_TH and *X. stockiae* bLPA18.4_TH therefore also need further investigation for a better understanding of the mode of action of the bioactive compounds.

The advantage of the use of the bacteria studied here is rapidly killing *Aedes* spp. larvae within 48 h; these also may be non-toxic to humans. Comparing to other bio-larvicides, *Aedes* spp. may develop resistance to *Beauveria bassiana*, *Metarhizium anisopliae* and *Wolbachia* [48–50]. *Aedes* spp., can escape copepod predation [51, 52]. *Bacillus thuringiensis* is more effective on the first and second larval stage than on the third and fourth larval stage of *Aedes* spp. but *Xenorhabdus* and *Photorhabdus* may be effective on all stages of *Aedes* larvae.

## Conclusions

In summary, 60 isolates of symbiotic bacteria from EPNs from upper northern Thailand consisted of 32 *Xenorhabdus* and 28 *Photorhabdus* isolates. The common species in the study area were *X. stockiae*, *P. luminescens akhurstii* and *P. luminescens hainanensis* while lower numbers of isolates of *X. miraniensis*, *X. ehlersii* and *P. luminescens laumondii* were observed. Three novel symbiotic associations identified included *P. luminescens akhurstii* - *H. gerrardi*, *P. luminescens hainanensis* - *H. gerrardi* and *X. ehlersii* - *S. scarabaei* which are of importance to our knowledge of the biodiversity of, and relationships between, EPNs and their symbiotic bacteria. Based on our bioassay, *Xenorhabdus* and *Photorhabdus* isolates are lethal to *Ae. aegypti* larvae. Different species and strains of symbiotic bacteria caused different levels of mortality in *Ae. aegypti*. *Xenorhabdus ehlersii* bMH9.2_TH begins to kill *Ae. aegypti* larvae within 48 h and is the best pathogen to the larvae. This indicates that *X. ehlersii* may be an alternative biological control agent for *Ae. aegypti* and other mosquitoes. The bioactive compounds in *X. ehlersii* should be investigated for further understanding of the application of these bacteria and their bioactive derivatives to the bio-control of mosquitoes.

## Abbreviations

CFU/ml: Colony forming unit/ml
DDT: Dichlorodiphenyltrichloroethane
DF: Dengue fever
EPN: Entomopathogenic nematode
LB: Luria-Bertani
NBTA: Nutrient bromothymol blue -triphenyltetrazolium chloride agar
OP: Organophosphates
*Pir*AB: *Photorhabdus* insect related AB
*rec*A: recombinase A
TSA: Tryptone soy agar
